# Characterizing Internet Searchers of Smoking Cessation Information

**DOI:** 10.2196/jmir.8.3.e17

**Published:** 2006-09-19

**Authors:** Nathan K Cobb, Amanda L Graham

**Affiliations:** ^2^Brown Medical SchoolDepartment of Psychiatry and Human BehaviorProvidenceRIUSA; ^1^Massachusetts General HospitalBostonMAUSA

**Keywords:** Smoking, cessation, Internet, search engine, query

## Abstract

**Background:**

The Internet is a viable channel to deliver evidence-based smoking cessation treatment that has the potential to make a large population impact on reducing smoking prevalence. There is high demand for smoking cessation information and support on the Internet. Approximately 7% (10.2 million) of adult American Internet users have searched for information on quitting smoking. Little is known about these individuals, their smoking status, what type of cessation services they are seeking on the Internet, or how frequently these searches for cessation information are conducted.

**Objective:**

The primary goal of this study was to characterize individuals who search for smoking cessation information on the Internet to determine appropriate triage and treatment strategies. The secondary goal was to estimate the incidence of searches for cessation information using publicly available search engine data.

**Methods:**

We recruited individuals who clicked on a link to a leading smoking cessation website (QuitNet) from within the results of a search engine query. Individuals were “intercepted” before seeing the QuitNet home page and were invited to participate in the study. Those accepting the invitation were routed to an online survey about demographics, smoking characteristics, preferences for specific cessation services, and Internet search patterns. To determine the generalizability of our sample, national datasets on search engine usage patterns, market share, and keyword rankings were examined. These datasets were then used to estimate the number of queries for smoking cessation information each year.

**Results:**

During the 10-day study period, 2265 individuals were recruited and 29% (N = 655) responded. Of these, 59% were female and overall tended to be younger than the previously characterized general Internet population. Most (76%) respondents were current smokers; 17% had quit within the last 7 days, and 7% had quit more than 7 days ago. Slightly more than half of active smokers (53%) indicated that they were planning to quit in the next 30 days. Smokers were more likely to seek information on how to quit and on medications; former smokers were more interested in how to cope with withdrawal. All participants rated withdrawal information and individually tailored information as being more useful, while displaying little interest in telephone counseling, expert support, or peer support. Publicly available data from large search engines suggest that 4 million Americans search for resources on smoking cessation each year.

**Conclusions:**

This study adds to the limited data available on individuals who search for smoking cessation information on the Internet, supports the prior estimates of the size of the population, and indicates that these individuals are in appropriate stages for both active cessation interventions and aggressive relapse prevention efforts. Continued development and evaluation of online interventions is warranted, and organizations seeking to promote cessation should carefully evaluate the Internet as a possible modality for treatment and as a gateway to other traditional programs.

## Introduction

The Internet has become the first source of health information for many people, primarily due to the ease of finding information [1]. In particular, there appears to be great demand for online information and services related to smoking cessation. In a random-digit dial survey conducted in 2004, 7% of Internet users in the United States reported using the Web to search for information on “how to quit smoking” [2]; more women reported to have looked then men (10% vs 7%), and unlike other health-related information seekers, they tended to be younger. At the time, this represented approximately 10.2 million people who had ever turned to the Internet for smoking cessation–related information or services. Little is known about these individuals, including their basic demographic characteristics, smoking status (eg, current smokers seeking cessation treatment, recent quitters seeking support to maintain abstinence), readiness to quit, quitting history, and treatment preferences. With the proliferation of antismoking sentiments and restrictive smoking policies, a diverse group of individuals may be turning to the Internet for assistance. In order to provide individually tailored and effective cessation treatment services via the Internet, it is necessary to better understand the characteristics and needs of this population.

The Internet is a powerful delivery channel that has the potential to deliver behavior change interventions on a population-wide basis to help people modify risk factors such as smoking [3]. There are limited, but encouraging, data to indicate that Web-based cessation interventions are effective in controlled trials [4-6]. However, it is not known if these approaches are appealing to or appropriate for the broader population of Internet users seeking cessation assistance. For example, approximately 30% of visitors to a widely utilized smoking cessation website indicated that they had quit smoking within the past week [7]. These individuals would be excluded from most randomized clinical trials of smoking cessation treatment, but they may represent a sizable population in need of assistance to remain abstinent. Information and services may need to be specially tailored to address the unique needs of individuals searching for cessation information based on their smoking status, demographic characteristics, and quitting history.

The incidence of cessation-related Internet searches may provide an effective proxy for consumer demand for cessation services. To date, there is little information about the rate at which searches for smoking cessation information occur. Several different techniques have been used to estimate the frequency of general health-related Internet searches [8-10], with widely varying results. Analyzing the first 300 search terms of the Wordtracker Top 500 keyword list, Phillipov and Phillips found less than 1% to be health-related terms [10]. Eysenbach took repeated snapshots of current search terms used on a search engine over a 15-month period, analyzed a random subset of queries, and found that 3.6-5.3% could be classified as health related [8]. Fox found that 79% of surveyed individuals had ever searched for health or medical information, while 7% had searched for smoking cessation information [2].

The primary purpose of this study was to characterize individuals who search for smoking cessation information. Specifically, we sought to gather information about sociodemographic and smoking history variables, search patterns (eg, time of day, search terms used), and perceptions about specific types of cessation services. Additionally, we used publicly available data to estimate the incidence of these searches. This information will be critical to develop appropriate and effective online cessation treatment programs, to triage patients as part of a stepped-care treatment model, or to successfully recruit smokers into treatment via the Internet.

## Methods

### Recruitment and Eligibility

Our recruitment strategy leveraged the prominent position of QuitNet (www.quitnet.com) on three of the largest Internet search engines. QuitNet is an established smoking cessation website [7] that is highly utilized, with over 600000 visitors and 97000 new registrants in 2004 from the United States alone. During the period of this study, it was listed in the top results for queries using “quit smoking” or “stop smoking” on three large search engines: Google, Yahoo!, and MSN (Appendix 1). In 2003, approximately 210000 (globally) and 110000 US individuals looking for information on quitting smoking arrived at QuitNet via these search engines. It has been estimated that 80% or more of Web users seeking health information start from search engines [11,12]. Research shows that Internet users read search engine results linearly, pay the most attention to the top three to five results, and click on the first promising link they find in the results [11,13]. Therefore, individuals who click on the link to QuitNet from a search engine results page are likely to be a representative sample of those individuals looking for cessation information on the Internet.

We recruited individuals based on four inclusion criteria: (1) use of the terms “quit smoking,” “quitting smoking,” “stop smoking,” or “stopping smoking” in a search engine query; (2) use of one of three major search engines (Google, Yahoo!, or MSN) to conduct these queries; (3) no prior visit to the QuitNet website (defined as www.quitnet.com or www.quitnet.org) as determined by the absence of a persistent (long-term) tracking cookie; and (4) location within the United States as determined by reverse lookup of IP (Internet protocol) addresses. When eligible Internet users clicked on the QuitNet link in the results of a search engine query, they were “intercepted” and recruited to participate in the study. The recruitment screen contained links to the survey and to the QuitNet website (Appendix 2). Those who accepted the invitation were directed to the QuitNet website following completion of the survey. Those who declined the survey invitation went directly to the QuitNet website. Recruitment for the survey was conducted for a total of 10 days: it began December 30, 2003, was suspended January 1 through January 3 due to technical concerns, and was completed January 12, 2004.

Generalizabilty was established from the complete panel of respondents, while we restricted further analysis to the respondents that reported any history of smoking and were seeking assistance for themselves.

### Measures

The survey consisted of 10 questions that included basic demographic information (age, gender), reasons for searching for cessation information, current smoking status, readiness to quit, quitting history (number of past quit attempts, length of quit, quit methods used), information desired, and ratings of perceived helpfulness of various online cessation features (eg, bulletin board, assistance in setting a quit date). The survey questions were administered on three separate screens, with no more than three questions per screen. Date and time of survey completion were automatically logged to the database.

Data on utilization of QuitNet after survey administration were extracted, including registration and total time online. Time online was defined as the time between the first page view after completion of the survey through the time of the last page view.

### Statistical Analyses

To determine the generalizability of our final sample, we compared survey respondents to nonrespondents who went on to register and use the QuitNet website on the demographic, website utilization, and search pattern variables obtained from the QuitNet database. In addition, we sought to determine the generalizability of our sample to the broader population of individuals who search for online smoking cessation information throughout the year. To do this, we examined the percentage of participants referred from each search engine as well as the total volume of cessation search terms used in Internet search engine queries, using publicly available data from Nielsen/NetRatings [14], Overture, and Wordtracker. Chi-square analyses were used to compare our sample to these national datasets.

For the 10-item survey, frequency tables were used to summarize the categorical data, and nonparametric tests were used to determine the statistical significance level. We used *t* tests for normally distributed continuous and ordinal variables.

Finally, to estimate the incidence of cessation-related Internet searches each year, we replicated the technique used by Eysenbach and Kohler [8]. MetaSpy was queried several times per day over the course of 9 months and the active queries were logged. Duplicate results (defined as the same set of 10 search terms being returned in succession) were removed. Searches containing the key words “quit[ing] smoking” or “stop[ing] smoking” were classified as cessation related.

## Results

### Recruitment Outcomes

**Figure 1:**
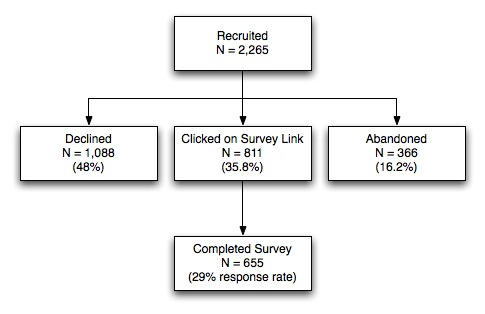
Eligibility and Recruitment Results

During the 10-day study period, 2265 eligible US residents were intercepted. Of those, 35.8% (N = 811) clicked on the “survey” link, 48% (N = 1088) clicked on the link to take them directly to the site (“declined”), and 16.2% (N = 366) did neither (“abandoned”). Of the 811 individuals who clicked through to the survey, 87.2% (N = 655) completed the full survey, yielding an overall response rate of 29% (Figure 1). Of the survey completers, 29 individuals reported having never smoked, leaving a final sample of 626 respondents.

### Generalizability

To assess generalizability, we compared all survey participants (N = 655) with nonrespondents who proceeded to register with QuitNet (N = 243). Overall, nonrespondents (N = 1454, abandoned and declined) were significantly less likely than survey respondents to register on QuitNet (16.7 vs 51.4%, Χ^2^_2_ = 303.7, *P* < .001). Compared to survey respondents, nonrespondents spent less time on QuitNet (4.5 vs 12.0 minutes, *t* = 13.4, *P* < .001) and viewed fewer pages (5.9 vs 15.3 pages, *t* = 16.0, *P* < .001) on the website. Nonrespondents were more likely to be female (59.4 vs 51.9%, Χ^2^_2_ = 4.2, *P* = .02) but did not differ by age, smoking status, time of survey invitation, or specific search engine used.

As shown in Table 1, the relative volume of participants referred from each search engine was consistent with national usage patterns (Χ^2^_2_ = 1.06, *P* = .59). In this study, 57% of participants were referred from Google, 29% from Yahoo!, and 14% from MSN. At the time of this study, 60% of all Internet search queries were estimated to be conducted using Google, 23% with Yahoo!, and 17% with MSN [14].

**Table 1:** Comparison of search engine usage to Nielsen/NetRatings statistics

	**Relative Reach of Search Engines**	
**Search Engine**	**Survey Recruitment (%)**	**National Usage (%)**
Google	57	60
Yahoo!	29	23
MSN	14	17
Total	100	100

The use of key search terms (“quit smoking,” “quitting smoking,” “stop smoking,” or “stopping smoking”) by survey respondents was also consistent with search patterns captured by Overture and Wordtracker. As shown in Table 2, the most commonly used search term was “quit smoking,” which constituted 52.9% of study queries, 59.1% of Overture queries, and 47.8% of Wordtracker queries. “Stop smoking” was the second most frequently used search term, which constituted 24.9% of study queries, 31.1% of Overture queries, and 36.5% of Wordtracker queries.

**Table 2:** Frequency of smoking-related search terms in search engine queries

**Search Term**	**Searches (%)**	**Survey Participants (%)** **(**Χ**^2^_4_ = 3.35, *P* = .80)**	**Overture (%)** **(**Χ**^2^_4_ = 152, *P* < .001)**	**Wordtracker (%)** **(**Χ**^2^_4_ = 138, *P* < .001)**
quit smoking	52.9	55.4	59.1	47.8
stop smoking	24.9	23.9	31.1	36.5
quitting smoking	21.9	20.4	9.0	13.4
stopping smoking	0.3	0.4	0.6	1.8
giving up smoking	0.00	0.00	0.2	0.6

### Participant Characteristics

As shown in Table 3, the majority of study participants were female (61.2%, n = 383) and between the ages of 26 and 44 years (62.7%, n = 393); 18.7% (n = 117) were aged 18-25 years, 17.1% (n = 107) were aged 45-64, and less than 1% were 65 or older (n = 4) or under age 18 (n = 5). Adjusted to local time of the participant, more than half (53.4%) of search engine queries for cessation information occurred during work hours (8 am-5 pm), 26.6% occurred between 5-9 pm, and 20% occurred at night (9 pm-6 am).

Participants were asked the reason they were searching for smoking cessation information. The majority of survey respondents (90.1%, n = 590) indicated that they were looking for help or support for themselves; 5.6% (n = 37) were looking for general information; 3.4% (n = 22) were looking for help for someone else; and 1% (n = 6) were health professionals or researchers looking for information. Further analyses were limited to individuals looking for cessation help or support for themselves or for general cessation information (N = 626). Among these individuals, 75.4% (n = 472) were current smokers, 17.4% (n = 109) had quit within 7 days (“recent quitters”), and 7.2% (n = 45) had quit more than 7 days ago (“longer-term quitters”).

**Table 3:** Demographic and smoking characteristics of study participants (N = 626)

**Characteristic**	**Number of Participants (%)**
**Age**	
< 18	5 (0.8)
18-25	117 (18.7)
26-34	232 (37.0)
35-44	161 (25.7)
45-54	87 (13.9)
55-64	20 (3.2)
65 or older	4 (0.6)
**Gender**	
Male	243 (38.8)
Female	383 (61.2)
**Smoking Status**	
Current smoker	472 (75.4)
Not thinking of quitting	1 (0.2)
Thinking of quitting in 6 months	222 (35.5)
Thinking of quitting in 30 days	249 (39.8)
Quit ≤ 1 week	109 (17.4)
Quit > 1 week, ≤ 1 month	43 (6.9)
Quit > 1 month	2 (0.3)

The majority of current smokers (52.8%, n = 249) planned to quit in the next 30 days, 47.0% (n = 222) planned to quit in the next 6 months, and one person (0.2%) was not thinking about quitting. Smokers had made an average of 5.1 quit attempts (SD = 14.7; median = 1) during the past year.

### Information Preferences

As shown in Table 4, information preferences varied by smoking status. Current smokers were more likely than recent quitters and longer-term quitters to be interested in information about how to quit smoking (88.1%, 54.1%, and 40.0%, respectively; Χ^2^_2_ = 104.7, *P* < .001) and medication usage (30.7%, 5.5%, and 4.4%, respectively; Χ^2^_2_ = 41.0, *P* < .001). Not surprisingly, both recent quitters and longer-term quitters were more interested than current smokers in information about withdrawal (77.1%, 66.7%, and 59.7%, respectively; Χ^2^_2_ = 11.7, *P* = .003).

**Table 4:** Information sought by smoking status (N = 626)

**Information**	**Current Smoker (%)** **(n = 474)**	**Quit ≤ 1 Week (%)** **(n = 109)**	**Quit > 1 Week (%)** **(n = 45)**	**Χ^2^_2_**	*P* value^*^
How to quit	88.1	54.1	40.0	104.7	< .001
Medications	30.7	5.5	4.4	41.0	< .001
Alternative methods	57.6	16.5	17.8	77.3	< .001
Withdrawal	59.7	77.1	66.7	11.7	.003

^*^Current smokers are the reference group.

Note: Multiple responses were allowed, so total percentages within smoking category exceed 100%.

### Perceived Helpfulness of Cessation Services

Participants were also asked to rate the perceived helpfulness of various smoking cessation treatment interventions on a scale from 1 to 5, with 1 representing “very helpful” and 5 representing “not helpful at all.” As shown in Table 5, the three features that were rated most highly by all participants were (1) individually tailored information (mean = 1.90, SD = 1.18); (2) information on withdrawal (mean = 1.84, SD = 1.15); and (3) a meter that keeps track of personal data (mean = 2.14, SD = 1.37). The three features rated the lowest by all participants were (1) support from a telephone counselor (mean = 3.21, SD = 1.35); (2) email support (mean = 2.95, SD = 1.40); and (3) support from others (mean = 2.90, SD = 1.38). Ratings of perceived helpfulness varied according to smoking status. Current smokers rated information about medications, assistance in setting a quit date, and assistance in choosing a medication as more helpful than did recent quitters and ex-smokers. Support from others and information about withdrawal received higher ratings of perceived helpfulness from recent quitters and ex-smokers than from current smokers. As detailed in Table 6, information of withdrawal, individually tailored information, and tracking meters were rated as “helpful” or “very helpful” by over half of the participants, while telephone counseling was thought to be helpful by less than 30% of participants.

**Table 5:** Perceived helpfulness of Internet features by smoking status

**Feature**	**All Participants, Mean (SD)(N = 626)**	**Current Smokers, Mean (SD)(n = 472)**	**Quit ≤ 1 Week(n = 109)**		**Quit > 1 Week(n = 45)**	
			**Mean (SD)**	*P* value^*^	**Mean (SD)**	*P* value^*^
Information on withdrawal	1.84 (1.15)	1.90 (1.17)	1.67 (1.08)	.06	1.51 (0.75)	.04
Individually tailored information	1.90 (1.18)	1.88 (1.18)	2.00 (1.25)	.36	1.79 (0.95)	.62
A meter that keeps track of personal data	2.14 (1.37)	2.14 (1.37)	2.14 (1.42)	1.0	2.15 (1.31)	.97
Information on medication side effects	2.59 (1.38)	2.55 (1.38)	2.79 (1.34)	.11	2.54 (1.43)	.97
Assistance in choosing a medication product	2.72 (1.37)	2.61 (1.36)	2.97 (1.38)	.02	3.24 (1.24)	.007
Information on medications	2.72 (1.36)	2.62 (1.36)	2.97 (1.37)	.02	3.23 (1.23)	.007
Online, personal help from a professional	2.81 (1.38)	2.79 (1.40)	2.86 (1.29)	.67	2.88 (1.39)	.70
Ability to find buddies	2.82 (1.37)	2.87 (1.39)	2.74 (1.29)	.40	2.59 (1.34)	.22
Assistance in setting a quit date	2.83 (1.39)	2.69 (1.37)	3.25 (1.32)	< .001	3.39 (1.37)	.003
Support via chat, forums, or email	2.90 (1.38)	2.98 (1.39)	2.67 (1.35)	.04	2.57 (1.30)	.07
Additional information that arrives by email	2.95 (1.40)	2.91 (1.43)	3.06 (1.30)	.34	3.08 (1.26)	.49
Talking by phone with a professional counselor	3.21 (1.35)	3.17 (1.39)	3.32 (1.22)	.32	3.46 (1.29)	.20

^*^ *P* values compared to current smokers; *P* = ns for all comparisons between recent and long-term quitters.

Note: 1 = very helpful; 2 = helpful; 3 = somewhat helpful; 4 = not very helpful; 5 = not helpful at all

**Table 6:** Proportion of participants (N = 626) rating Internet cessation services as helpful or very helpful

**Feature Offered**	**Helpful or Very Helpful**	
	**n**	**%**
Information on withdrawal	460	73.5
Individually tailored information	450	71.9
A meter that keeps track of personal data	405	64.7
Information on medication side effects	303	48.4
Information on medications	275	43.9
Assistance in choosing a medication product	273	43.6
Online, personal help from a professional	265	42.3
Ability to find buddies	250	39.9
Assistance in setting a quit date	248	39.6
Support from others, via chat, forums, or email	233	37.2
Additional information that arrives by email	223	35.6
Talking by phone with a professional counselor	184	29.4

### Estimating Incidence of Cessation Queries

Over the course of 9 months, 541685 searches were extracted from MetaSpy, of which a total of 38 were smoking cessation related. Assuming a total search engine volume of 52 billion searches per year [14], this ratio yields an estimate of 3.6 million (99% CI = 2.5-4.8 million) cessation-related searches per year in the United States alone.

## Discussion

The Internet holds great potential to impact population smoking prevalence by delivering evidence-based treatments to greater numbers of smokers who may never receive treatment through other modalities. This is the first study to characterize the population of individuals looking for cessation information online. Results suggest that the Internet may be an effective way to reach smokers who are younger, who search for cessation services during work hours, and who have recently quit on their own.

The relatively large proportion (17.4%) of recent quitters (within 7 days) in this study who are actively seeking assistance is of particular importance. The majority of self-quitters relapse within 8 days [15]. Over 16 million Americans try to quit on their own each year, but less than 5% maintain abstinence for 3 months [16]. Thus, more than 15 million smokers relapse. Until recently [17], this segment of the population of smokers received little attention once formal cessation treatments ended. Given the reach and 24/7 availability of the Internet, effective relapse prevention interventions can and should be delivered to the thousands of smokers trying to maintain abstinence. An effective relapse prevention service for self-quitters with intensive support around the quit date could produce a significant impact on smoking prevalence and could be used in conjunction with any other cessation treatment.

New population-based strategies to identify and reach smokers with evidence-based cessation treatment are needed [3]. Currently, telephone quit lines are the primary public health delivery channel for low cost, effective tobacco treatment. Despite the obvious advantages of convenience and cost, uptake rates in states with quit lines have remained low despite aggressive promotion, with less than 2% of smokers participating [18]. Given that Internet searchers are more likely to prefer self-help treatment with lower efficacy rates, it is important to design interventions which capture initial interest that can successfully “up-sell” more intensive and effective treatment interventions such as telephone counseling and medication use. In this manner, the Internet may be able to provide a workable model for stepped care, where participants can be further triaged to receive telephone counseling; prescription medication; in-person, group, or individual counseling; or even inpatient treatment [19].

### Limitations

Several limitations should be considered when interpreting results of this study. The relatively low response rate (29%) raises concern about the generalizability of findings. Survey respondents were more likely to go on to register with the site; this likely indicates that they were in a more advanced stage of change than nonrespondents. It may, however, also indicate that the survey itself acted as an incentive to proceed to registration. Furthermore, we worked from the assumption that individuals who clicked on the link to QuitNet in search engine results were representative of the entire population of searchers. Although consistent with utilization patterns of search engines, this assumption has never been tested for searches on smoking cessation, or the QuitNet site in particular. It is possible that less motivated searchers may find the query results unappealing and not click on any link at all, thus biasing our results toward individuals closer to quitting.

A second potential limitation is the method we used to estimate the total number of people seeking smoking cessation information each year. This method does not take into account searches using other keywords or individuals using resources other than search engines to find information (eg, health Web portals, referrals from health professionals, direct-to-consumer advertising, or quit lines). In addition, individuals may search for information multiple times, making it difficult to estimate the actual number of unique individuals as opposed to the total number of searches. Finally, the dataset used to derive these estimates is of commercial nature and published online in a promotional context. It has not been peer-reviewed or made available in its raw form. The data for this study were collected from 2003-2004; it is possible that in the intervening time the demographics or search behavior of smokers has changed. However, given the limited changes in both search engine technology as well as the demographics of smokers in the United States, this seems unlikely. Despite these limitations, this study provides valuable information about people who search for smoking cessation information online, and it demonstrates a new methodology for validating this kind of survey data.

### Conclusion

This study suggests that the potential public health impact that can be achieved through Internet-based smoking cessation programs is significant given the reach of the Internet—should these interventions be proven effective. Given that individuals may conduct multiple searches, our estimate of 3.6 million active searches per year for smoking cessation information is consistent with the 2004 data that showed 7% (about 10 million) of Internet users in the United States had searched for information on quitting smoking [2]. With 1.25 billion smokers throughout the world [20], there is enormous potential to globally impact smoking prevalence.

The public health community has invested heavily over the past 15 years in successfully de-normalizing smoking and encouraging cessation. However, low uptake rates seen in clinical programs and telephone quit lines call for new population-based approaches. Even if Internet-assisted tobacco interventions prove to have limited efficacy, the Web may still serve as a point of entry to multi-modality treatment programs. These programs may serve to simply link online searchers to more traditional treatment programs (such as telephone counseling or local group sessions), provide pharmaceutical products, or, in more sophisticated settings, use the Web as a platform to integrate voice counseling, local groups, mailed pharmaceutical products, and other proven modalities. We anticipate that the consumer demand demonstrated in this report will ultimately drive increasing services that will reflect a mixture of these different evidence-based treatments.

## Figures and Tables

**Figure 2:**
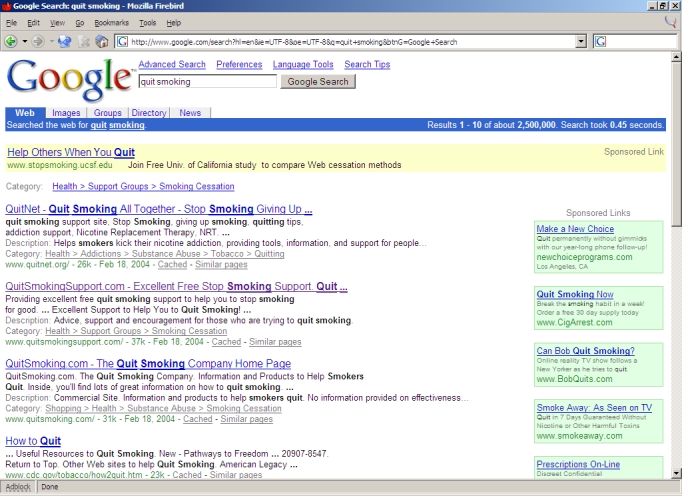
Sample search engine screenshot

**Figure 3:**
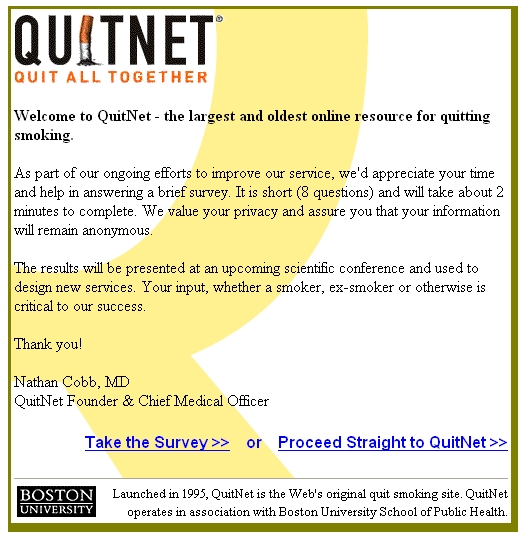
Survey invitation interception page
